# Laparoscopic assisted colectomy versus laparoscopic complete colectomy: a cost analysis

**DOI:** 10.1007/s13304-024-01876-6

**Published:** 2024-05-17

**Authors:** Zhaoyang Zheng, Qiang Du, Libin Huang, Lie Yang, Zongguang Zhou

**Affiliations:** 1 Division of Gastrointestinal Surgery, Department of General Surgery, West China Hospital of Sichuan University, No. 37 Guoxue Lane, Chengdu, 610041 Sichuan China; 2 Institute of Digestive Surgery, State Key Laboratory of Biotherapy and Cancer Center, West China Hospital Sichuan University, No. 37 Guoxue Lane, Chengdu, 610041 Sichuan China

**Keywords:** Colon cancer, Intracorporeal anastomosis, Cost analysis, Propensity score matching

## Abstract

To compare the short-term outcomes and explore the potential economic benefits of laparoscopic-assisted colectomy with extracorporeal anastomosis (LAC/EA) vs. laparoscopic complete colectomy with intracorporeal anastomosis (LCC/IA) for patients with non-metastatic resectable colon cancer. Data of patients who underwent laparoscopic hemicolectomy from January 2017 to March 2023 were collected and analyzed. Propensity score matching (PSM) analyses was carried out to minimize the selection bias. Before PSM, a total of 113 patients met the inclusion criteria (39 in the LCC/IA vs. 74 in the LAC/EA). Clinicopathologic characteristics were comparable except for the median number of removed lymph nodes (*P* = 0.023). LCC/IA was associated with longer operative time, less intraoperative blood loss, and shorter incision length. The rate of 30-day postoperative complications was similar, but the time to first flatus and soft diet was shorter in the LCC/IA. No deaths were reported in either group within 30 days after surgery. Costs of surgical instruments (25,945.8 ± 1,918.0 vs. 23,551.9 ± 2,665.5 RMB; *P* < 0.01) were higher for the LCC/IA but overall costs were similar (LCC/IA, 43,220.0 ± 4,954.0 vs. LAC/EA, 41,269.2 ± 6,685.9 RMB; *P* = 0.112). After PSM, 38 patients in the LCC/IA and 63 patients in the LAC/EA were compared. LCC/IA was superior in terms of intraoperative blood loss, incision length, and postoperative functional recovery. There was an extra charge of 2385.0 RMB regarding surgical instruments in the LCC/IA but the overall cost did not reach statistical significance. LCC/IA is a feasible, safe, and cost-effective surgical treatment for patients with non-metastatic resectable colon cancer.

## Introduction

Since the introduction of laparoscopic colectomy in 1991 [1], minimally invasive surgery has become the dominant option for colorectal cancer. Laparoscopy has been recommended by NCCN guidelines as the surgical choice for patients with non-metastatic resectable colon cancer. RCTs such as COLOR [2]. and CLASICC [3] have demonstrated the advantages of laparoscopic colectomy over open surgery, including earlier bowel functional recovery, shorter hospital stay, less postoperative morbidity and comparable oncological outcomes [4–7]. Following laparoscopic colectomy, the anastomosis can be created using an extracorporeal or intracorporeal approach. As the primary curative treatment, laparoscopic-assisted colectomy with extracorporeal anastomosis (LAC/EA) is used more frequently. After routine mesenteric mobilization and vessel ligation laparoscopically, the bowel is extracted through a minilaparotomy in the abdomen to perform the anastomosis. However, the disadvantages of LAC/EA include the risk of bowel traction/twisting, implantation metastasis and hemorrhage due to bowel exteriorization. Therefore, laparoscopic complete colectomy with intracorporeal anastomosis (LCC/IA) has been applied as an alternative method of anastomosis. This technique could compensate for these drawbacks, offer more options to determine the location for specimen traction and enable great lymph node yield achievement [8, 9]. Despite these benefits, LCC/IA remains a less commonly used technique because of its inherent technical difficulties and potential risks such as intraoperative contamination and tumor exposure [7]. Current researches regarding LCC/IA have mainly focused on the clinical results and long-term prognosis; however, the economic benefits has been infrequently studied. Thus, it is still unclear which anastomosis technique (extracorporeal or intracorporeal) is more cost-effective. Given the fact that colorectal cancer has now ranked as the second leading cause of cancer-related deaths worldwide, the healthcare system is facing a growing economic strain. Hence, there is a crucial need to explore an economically efficient laparoscopic surgical approach to optimize outcomes for patients with colonic malignancy.

The purpose of our study is to compare the short-term outcomes and explore the potential economic benefits of LCC/IA versus LAC/EA for non-metastatic resectable colon cancer. We set postoperative functional recovery as the primary endpoint. Our secondary endpoint was to conduct a cost analysis by evaluating cost parameters. Propensity score matching (PSM) analyses were used to minimize the selection bias.

## Materials and methods

### Patient selection

This is a non-randomized, retrospective study involving clinical and economic analysis in a single institution. Approval was granted by the Biomedical Ethics Review Committee of West China Hospital of Sichuan University (2023–652) and informed consent was obtained from the participants. Data of patients who underwent laparoscopic hemicolectomy at the Division of Gastrointestinal Surgery, Department of General Surgery in West China Hospital, Sichuan University from January 2017 to March 2023 were collected and compared.

Inclusion criteria: (1) 18 to 85 years old; (2) Adenocarcinoma diagnosis confirmed by colonoscopy and pathology before surgery.

**Fig. 1:**
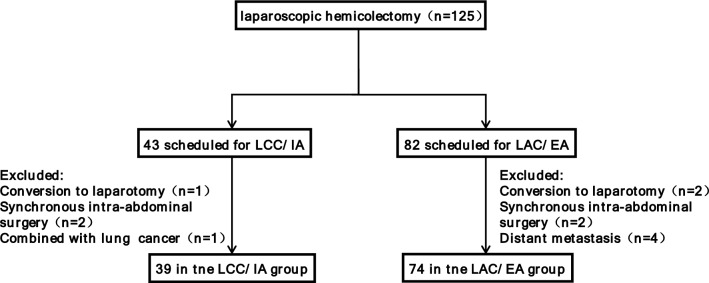
Patient selection flow

Exclusion criteria: (1) Preoperative evidence of adjacent organs invasion or distant metastasis; (2) Conversion to laparotomy; (3) Emergency surgery due to obstruction, perforation or bleeding resulting from colon; (4) Synchronous intra-abdominal surgery (cholecystectomy, gastrectomy, splenectomy, etc.); (5) Combination with other incurable malignancy; (6) Medical records were incomplete or missing. During the period 125 patients received laparoscopic hemicolectomy and 113 patients were eligible for analysis. The patient selection flow is presented in Fig. 1.

### Surgical procedures

Patients were assigned to two groups according to different anastomotic methods: LCC/IA or LAC/EA. All enrolled patients were operated by a single experienced surgeon in a single medical center.

For patients scheduled for elective surgery, if the patient was in the absence of typical obstructive symptoms (pain, distension, obstruction, or vomiting), postprandial bloating, or difficulty in eating or passing stool due to bloating. Mechanical bowel preparation (polyethylene glycol) was implemented 1 day before surgery. However, if the patient had obstructive symptoms, or bloating sensation after food intake, difficulty in eating or passing stool because of bloating. Mechanical bowel preparation started 24 h before surgery may cause fluid accumulation and dilation of the proximal colon (including the small intestine), which could impact laparoscopic manipulation and increase the risk of abdominal contamination as well as surgical site infection. In that case, mild oral laxative was ingested 3 days before surgery and the right dose of polyethylene glycol was ingested 2 days before the operation. Oral antibiotics were routinely used 1 day before surgery.

### Laparoscopic complete colectomy with intracorporeal anastomosis (LCC/IA)

**Fig. 2:**
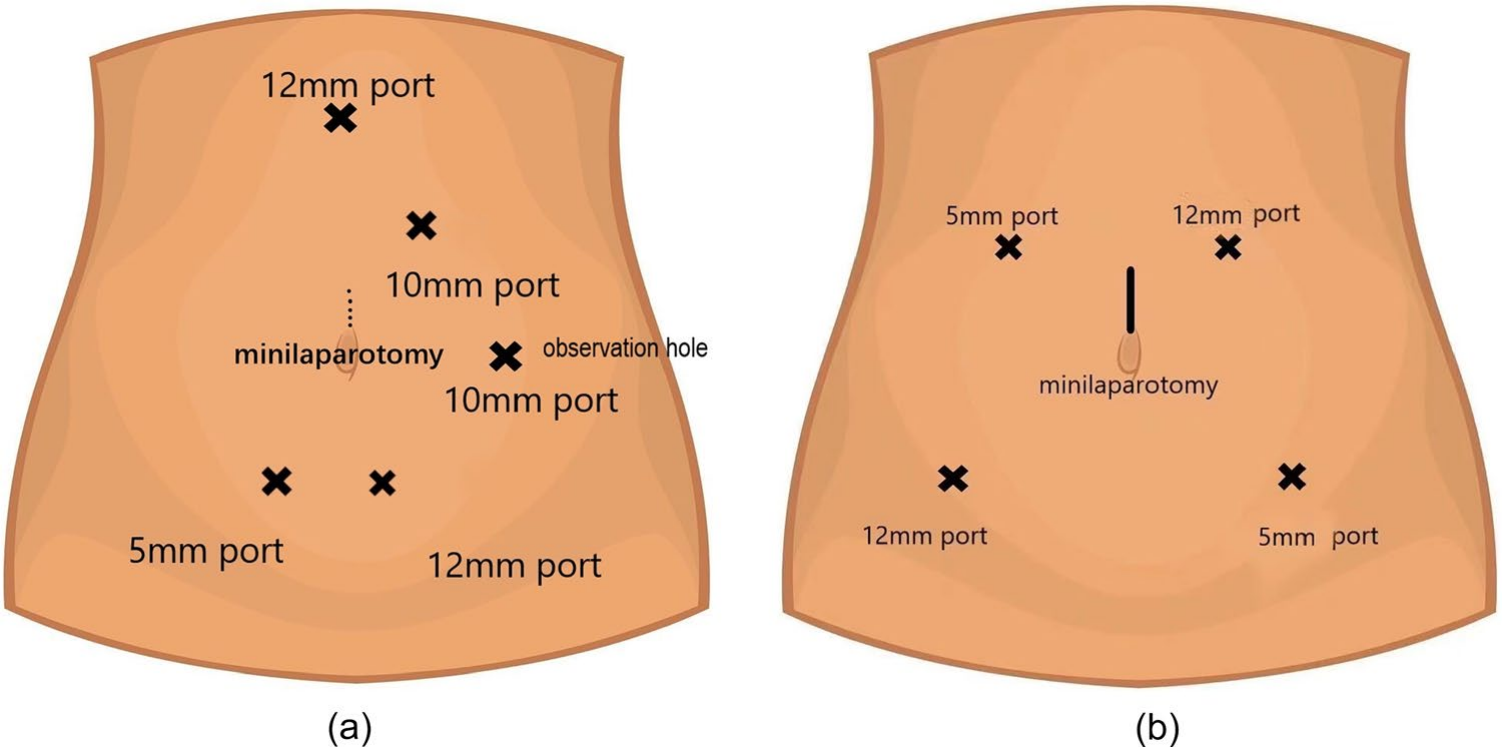
Distribution of trocars of laparoscopic complete colectomy. **a** Laparoscopic complete right hemicolectomy. **b** Laparoscopic complete left hemicolectomy

Taking right hemicolectomy as an example, the patient was placed in a supine position with both legs kept in abduction after general anesthesia and pneumoperitoneum establishment. The surgeon stood between the patient's legs, while the camera holder and an assistant stood on the patient's left side. We applied a five-port laparoscopic approach [11]. Two working ports were symmetrically distributed on both sides of the suprapubic region with 12-mm and 5-mm trocars. One of the assistant's ports was placed on the left hypochondrium with a 10-mm trocar, and the other was placed below the xiphoid with a 12-mm trocar. The camera port (observation hole) was placed on the left side of the umbilicus with a 10-mm trocar. The right colon and mesentery were mobilized according to the medial-to-lateral approach along Toldt’s gap. Then, after completing the mesenteric separation of the planned bowel segments, we dissected the lymph nodes and performed ligation of the ileocolonic vessels, right colonic vessels, and right branches of the middle colonic vessels at the roots of these vessels. The distribution of trocars of total laparoscopic colectomy is shown in Fig. 2.

**Fig. 3:**
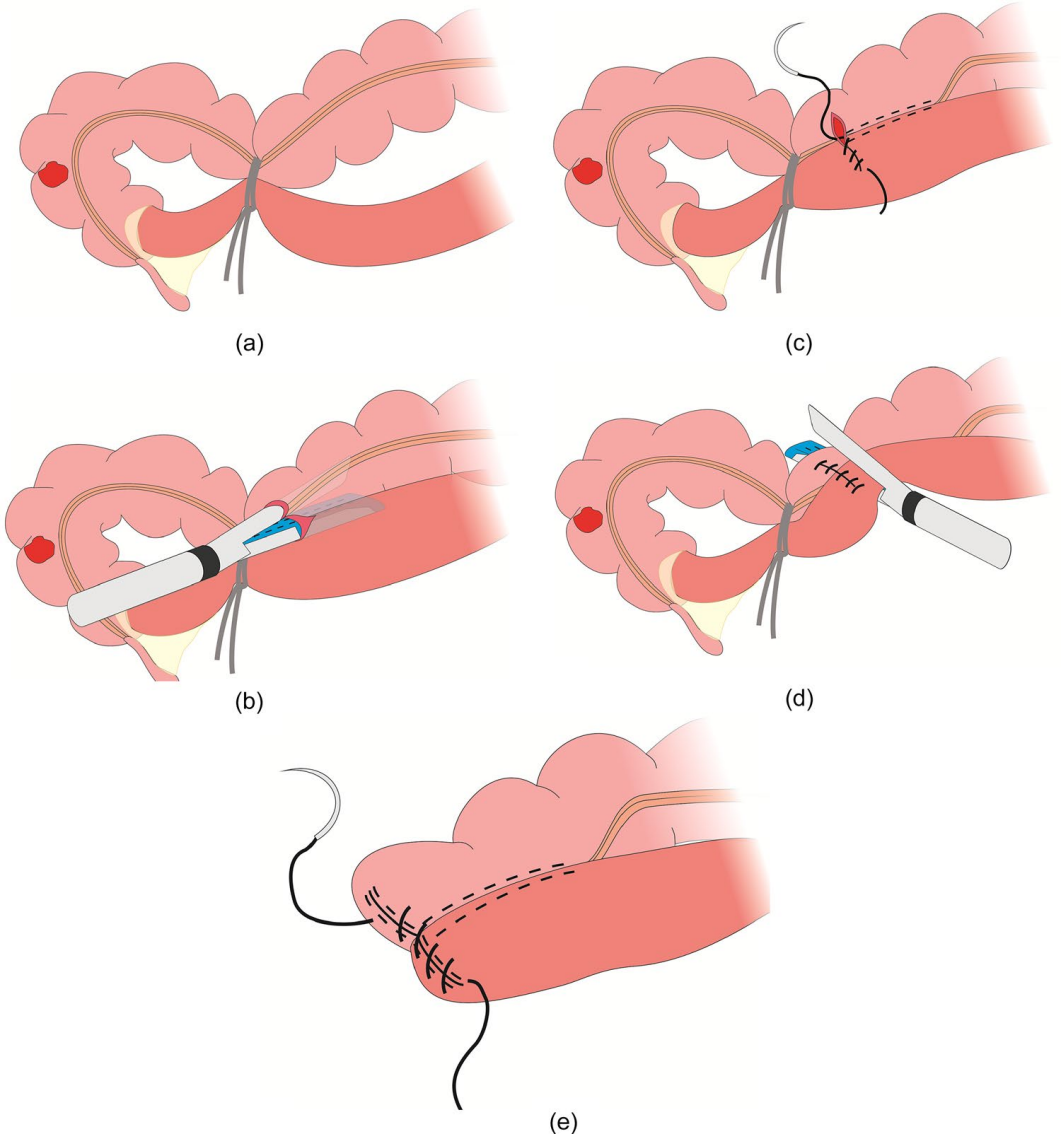
Process of UEEA approach^*^. **a** The bowels are tied together into a U-shape by a ligature. **b** Anastomosis is fashioned using a linear stapler. **c** The common opening is tightened with a suture. **d** Bowel transection and stump closure using the stapler. **e** The stump is reinforced by barbed wire. ^*^These figures are quoted from [43] and we have obtained permission from the copyright owners

Our team performed intracorporeal anastomosis via the “U-tied functional end-to-end anastomosis (UEEA)” approach, which was described in our previous study [11, 12]. For UEEA anastomosis: (1) Aligning the proximal and distal bowel in an antiperistaltic configuration with a ligature and then the specimen was tied into a U-shape; (2) Making enterotomies on both sides of the antimesenteric region of the bowel segments, and the first anastomosis was completed by a 60-mm endoscopic linear stapler (Echelon Flex 60, PSEE60A, Ethicon Endo-Surgery, LLC, Guaynabo, Puerto Rico, USA). The anastomosis was not routinely reinforced unless under specific circumstances, such as bleeding. (3) Tightening the common opening using a suture, then bowel transection and stump closure were completed simultaneously by the stapler; (4) Reinforcing the stump with 3–0 barbed wire, especially at the T-cross of the staples. (5) Removing the specimen through a minilaparotomy under the protection of a specimen sleeve. Figure 3 illustrates the specific process of the procedure.

For left hemicolectomy, patients used the same supine position and a five-port laparoscopic approach was utilized. The detailed description of trocar distribution was described in [12]. After the establishment of pneumoperitoneum, we mobilized the left colon and its mesentery using the previously mentioned “five-step process” [13]. Then the UEEA approach was performed. It is worth noting that the mobilization of the bowel should be at least 10 cm on both sides of the tumor to get sufficient free before performing UEEA anastomosis.

### Laparoscopic assisted colectomy with extracorporeal anastomosis (LAC/EA)

Laparoscopic right hemicolectomy was performed under a standardized approach [18]. The brief procedures were present as follows: (1) The terminal ileum, ascending colon, and proximal transverse colon were mobilized, the lymph nodes were dissected and the vessels were ligated laparoscopically. (2) Exteriorization of the bowel through a relatively big umbilical incision. (3) Division of the planned bowel segments and its mesentery were performed. (4) Ileocolic anastomosis was fashioned extracorporeally and the bowel was returned to the abdominal cavity after pneumoperitoneum re-establishment.

For laparoscopic left hemicolectomy, “five-step process” was applied. The specific surgical procedures can be found in [13].

### Data collection

All data included in this study were acquired retrospectively from our institutional prospective database and used for research purpose. 30-day postoperative complications, 30-day readmission/reoperation, and 30-day mortality were prospectively followed. The collected variables consisted of baseline characteristics, pathological characteristics, perioperative outcomes, and costs of hospitalization. Detailed information is elaborated below.

Baseline characteristics involving age, gender, body mass index (BMI), American Society of Anesthesiologists (ASA) score, preoperative comorbidity, history of previous abdominal surgery, tumor location were recorded. The Charlson-Age Comorbidity Index (CCI) score was used to assess preoperative comorbidity severity [14].

For perioperative outcomes, intraoperative results including operation type, anastomosis type, operative time, intraoperative blood loss and incision length were investigated. Metrics of postoperative functional recovery including time to first flatus and soft diet, pain scale, and postoperative length of stay (LOS) were selected. Other postoperative variables, including 30-day postoperative complications, mortality, and readmission/reoperation were also collected. We used visual analgesic scale (VAS) for pain evaluation at the first, second, and third postoperative day (POD), and Clavien–Dindo (CD) classification for the severity assessment of postoperative complications [15].

Pathological characteristics including number of resected lymph nodes, stage of tumor invasion, stage of lymph node invasion and pathological (UICC) stage were recorded.

Costs on hospitalization contain the following parameters: (1) Intraoperative costs: surgical instruments, anesthesia-related fee, intraoperative drugs prescribed by anesthetists and surgeons; (2) Perioperative costs: diagnostic tools including radiographic examinations, pathological examinations, microbial and laboratory analyses, ward fee including room and board and nursing charges, perioperative drugs including rehydration, antibiotics, nutraceuticals and blood products; (3) Overall costs during the hospital stay. All diagnosis and treatment costs are paid in Chinese Renminbi (RMB).

### Statistical analysis

Analysis was performed using the IBM SPSS Statistics for Windows, version 26.0 (IBM Co., Armonk, NY, USA). For continuous variables, mean ± standard deviation (*SD*) or median (interquartile range) were represented. Significant between-group differences were tested with Student *t*-test or non-parametric Wilcoxon rank sum tests, according to data distribution. For categorical variables, frequency (*N*) with percentage (*%*) were described. The analysis was carried using Chi-square test or Fisher exact probability test. Results were considered statistically significant when *P* < 0.05.

In order to control confounding variables between groups, propensity score matching (PSM) analysis was conducted by a logistic regression model. We set the anastomosis method (LCC/IA vs. LAC/EA) as the dependent variable. The samples are matched via “1:2 nearest neighbor matching”.

## Results

### Clinicopathologic characteristics

Before PSM, a total of 113 patients were enrolled (39 in the LCC/IA vs. 74 in the LAC/EA). After PSM analysis, the study population was composed of 101 patients (38 in the LCC/IA vs. 63 in the LAC/EA). No significant differences were detected in terms of clinical characteristics including demographics, preoperative physical condition, and history of previous abdominal surgery before or after PSM. Pathological characteristics were similar between the two groups except for the median number of removed lymph nodes (LCC/IA, 18(16–27) vs. LAC/EA, 16(13.8–22); *P* = 0.023). However, this covariate became balanced in PSM analysis. Table 1 and Table 2 listed detailed baseline and pathological characteristics.

**Table 1:** Patient characteristics before and after propensity score matching

	Before propensity score matching			After propensity score matching		
	LCC/IA(*N* = 39)	LAC/EA(*N* = 74)	*P* value	LCC/IA(*N* = 38)	LAC/EA(*N* = 63)	*P* value
Age, mean ± *SD*, years	60.4 ± 11.0	61.0 ± 12.4	0.832	60.1 ± 11.0	61.2 ± 14.0	0.672
Gender, *N* (%)			0.340			0.425
Male	19 (48.7)	43 (58.1)		18 (47.4)	35 (55.6)	
Female	20 (51.3)	31 (41.9)		20 (52.6)	28 (44.4)	
BMI, mean ± *SD*, Kg/m^2^	22.7 ± 2.7	22.7 ± 3.3	0.904	22.7 ± 2.7	22.7 ± 3.2	0.960
ASA score, *N* (%)			0.545			0.563
II	29 (74.4)	51 (68.9)		28 (73.7)	43 (68.3)	
III	10 (25.6)	23 (31.1)		43 (68.3)	20 (31.7)	
Comorbidity, *N* (%)						
Total	27 (69.2)	61 (82.4)	0.108	27 (71.1)	51 (81.0)	0.250
Cardiovascular	12 (30.8)	27 (36.5)		12 (31.6)	21 (33.3)	
Peripheral vascular	0	1 (1.4)		0	0	
Hematological	3 (7.7)	11 (14.9)		3 (7.9)	8 (12.7)	
Endocrine	2 (5.1)	7 (9.5)		2 (5.3)	4 (6.3)	
Pulmonary	4 (10.3)	15 (20.3)		4 (10.5)	14 (22.2)	
Digestive (liver/gastrointestinal)	12 (30.8)	39 (52.7)		12 (31.6)	33 (52.4)	
Obesity	1 (2.6)	4 (5.4)		1 (2.6)	4 (6.3)	
Others	^a^1 (2.6)	^b^2 (2.7)		^a^1 (2.6)	^b^2 (3.2)	
CCI score, *N* (%)			0.069			0.336
≤ 5	33 (84.6)	51 (68.9)		32 (84.2)	48 (76.2)	
> 5	6 (15.4)	23 (31.1)		6 (15.8)	15 (23.8)	
Previous abdominal surgery, *N* (%)	13 (33.3)	31 (41.9)	0.375	13 (34.2)	25 (39.7)	0.582
Tumor location, *N* (%)			0.306			0.389
Right colon	26 (66.7)	42 (56.8)		25 (65.8)	36 (57.1)	
Left colon	13 (33.3)	32 (43.2)		13 (34.2)	27 (42.9)	

^a^1 rheumatoid arthritis

^b^1 psoriasis and 1 syphilis

**Table 2:** Pathological characteristics before and after propensity score matching

	Before propensity score matching			After propensity score matching		
	LCC/IA(*N* = 39)	LAC/EA(*N* = 74)	*P* value	LCC/IA(*N* = 38)	LAC/EA(*N* = 63)	*P* value
No. of resected lymph nodes, *M*(IQR)	18 (16–27)	16 (13.8–22)	**0.023**	18(15.8–22.5)	16(14–22)	0.076
pT stage, * N* (%)			0.737			0.775
T0/Tis	2 (5.1)	3 (4.1)		2 (5.3)	2 (3.2)	
T1	3 (7.7)	6 (8.1)		3 (7.9)	4 (6.3)	
T2	5 (12.8)	5 (6.8)		5 (13.2)	5 (7.9)	
T3	19 (48.7)	44 (59.5)		18 (47.4)	37 (58.7)	
T4	10 (25.6)	16 (21.6)		10 (26.3)	15 (23.8)	
pN stage, * N* (%)			0.798			0.882
N0	28 (71.8)	48 (64.9)		27 (71.1)	41 (65.1)	
N1	9 (23.1)	21 (28.4)		9 (23.7)	18 (28.6)	
N2	2 (5.1)	5 (6.8)		2 (5.3)	4 (6.3)	
UICC stage, *N* (%) adenoma/in situ	2 (5.1)	3 (4.1)	0.617	2 (5.3)	2 (3.2)	0.534
I	8 (20.5)	9 (12.2)		8 (21.1)	7 (11.1)	
II	18 (46.2)	36 (48.6)		17 (44.7)	32 (50.8)	
III	11 (28.2)	26 (35.1)		11 (28.9)	22 (34.9)	

Bold value indicate the significant differences (p < 0.05) between groups

### Intraoperative outcomes

The intraoperative outcomes are summarized in Table 3. The two groups did not differ in the operation type; however, the anastomosis type was significantly different both before and after PSM (*P* < 0.01).

**Table 3:** Intraoperative outcomes before and after propensity score matching

	Before propensity score matching			After propensity score matching		
	LCC/IA(*N* = 39)	LAC/EA(*N* = 74)	*P* value	LCC/IA(*N* = 38)	LAC/EA(*N* = 63)	*P* value
Operation type,* N* (%)			0.249			0.330
Right colectomy	25 (64.1)	42 (56.8)		24 (63.2)	36 (57.1)	
Left colectomy	13 (33.3)	32 (43.2)		13 (34.2)	27 (42.9)	
Extended hemicolectomy	1 (2.6)	0		1 (2.6)	0	
Anastomosis type, *N* (%)			** < 0.01**			** < 0.01**
Stapled	39 (100.0)	60 (81.1)		38 (100.0)	51 (81.0)	
Manual + stapled	0	14 (18.9)		0	12 (19.0)	
Operative time, mean ± *SD*, min	226.8 ± 39.4	203.8 ± 45.9	** < 0.01**	227.9 ± 39.4	200.0 ± 43.3	< 0.01
Intraoperative blood loss, mean ± *SD*, ml	24.7 ± 23.5	36.4 ± 28.8	**0.033**	25.1 ± 23.7	37.0 ± 30.5	0.043
Incision length, mean ± *SD*, cm	4.9 ± 0.7	5.6 ± 0.7	** < 0.01**	5.0 ± 0.7	5.7 ± 0.7	< 0.01

Bold values indicate the significant differences (p < 0.05) between groups

Patients who underwent LCC/IA present significantly longer operative time (226.8 ± 39.4 vs. 203.8 ± 45.9 min; *P* < 0.01), less intraoperative blood loss (24.7 ± 23.5 vs. 36.4 ± 28.8 ml; *P* = 0.033) and shorter incision length (4.9 ± 0.7 cm vs. 5.6 ± 0.7 cm; *P* < 0.01). The differences were still present and reached significance following PSM analysis.

### Postoperative outcomes

Table 4 reported all the postoperative functional recovery and other postoperative parameters. LCC/IA showed significantly earlier first flatus both before (3.1 ± 0.8 vs. 3.8 ± 1.2 days; *P* < 0.01) and after PSM (3.2 ± 0.8 vs. 3.7 ± 1.1 days; *P* < 0.01). Similar results were also observed in start time for soft diet, both in the unmatched (3.8 ± 0.9 vs. 4.5 ± 1.4 days; *P* < 0.01) and PSM analyses (3.8 ± 0.9 vs. 4.4 ± 1.3 days;* P* < 0.01).

**Table 4:** Postoperative outcomes before and after propensity score matching

	Before propensity score matching			After propensity score matching		
	LCC/IA(*N* = 39)	LAC/EA(*N* = 74)	*P* value	LCC/IA(*N* = 38)	LAC/EA(*N* = 63)	*P* value
Time to first flatus, mean ± *SD*, days	3.1 ± 0.8	3.8 ± 1.2	** < 0.01**	3.2 ± 0.8	3.7 ± 1.1	** < 0.01**
Time to soft diet, mean ± *SD*, days	3.8 ± 0.9	4.5 ± 1.4	** < 0.01**	3.8 ± 0.9	4.4 ± 1.3	** < 0.01**
Pain scale, mean ± *SD*						
POD1	1.9 ± 0.6	2.0 ± 0.8	0.650	1.9 ± 0.5	1.9 ± 0.6	0.983
POD2	1.6 ± 0.6	1.8 ± 0.7	0.294	1.6 ± 0.6	1.8 ± 0.8	0.202
POD3	1.2 ± 0.7	1.5 ± 0.9	0.076	1.2 ± 0.7	1.5 ± 0.8	0.064
Postoperative LOS, mean ± *SD*, days	5.62 ± 1.16	6.35 ± 1.88	**0.012**	5.63 ± 1.17	6.33 ± 1.94	**0.026**
30-day mortality, *N* (%)	0	0	–	0	0	–
30-day postoperative complications, *N* (%)						
Total	2 (5.1)	8 (9.5)	0.658	2 (5.3)	6 (7.9)	0.914
Wound infection	1 (2.6)	1 (1.4)	1.000	1 (2.6)	0	–
Gastric retention	1 (2.6)	0	–	1 (2.6)	0	–
Pulmonary infection	0	1 (1.4)	–	0	1 (1.6)	–
Hematochezia	0	1 (1.4)	–	0	1 (1.6)	–
Chyle leakage	0	3 (4.1)	–	0	2 (3.2)	–
Bowel obstruction	0	1 (1.4)	–	0	1 (1.6)	–
Anastomotic leakage	0	1 (1.4)	–	0	1 (1.6)	–
Clavien–Dindo classification, *N* (%)			0.929			0.906
Grade I	1 (2.6)	3 (4.1)	–	1 (2.6)	2 (3.2)	–
Grade II	1 (2.6)	3 (4.1)		1 (2.6)	2 (3.2)	
Grade ≥ III	0	^a^2 (2.7)		0	^a^2 (3.2)	
30-day Readmission/Reoperation, *N* (%)	0	2 (2.7)	1.000	0	2 (3.2)	1.000

Bold values indicate the significant differences (p < 0.05) between groups

^a^1 anastomotic leakage and 1 bowel obstruction

Although the difference in pain scale did not achieve statistical significance, we noted that LCC/IA had lower pain VAS scores at POD3, both before (1.2 ± 0.7 vs. 1.5 ± 0.9; *P* = 0.076) and after propensity score matching (1.2 ± 0.7 vs. 1.5 ± 0.8; *P* = 0.064).

Mean postoperative LOS was significantly shortened by 0.7 day under LCC/IA approach both in the pre-PSM (5.62 ± 1.16 vs. 6.35 ± 1.88 days; *P* = 0.012) and post-PSM data (5.63 ± 1.17 vs. 6.33 ± 1.94 days; *P* = 0.026).

No deaths occurred within 30 days after the procedure and no differences were registered regarding severe complications (CD Grade ≥ III) between the two groups within 30 days of discharge both before and after PSM.

The incidence of 30-day postoperative complications, graded according to CD classification, showed comparable rates in both groups. However, LCC/IA group was associated with a lower overall rate, both pre- (5.1% vs. 9.5%, respectively; *P* = 0.658) and post-PSM analysis (LCC/IA, 5.3% vs. LAC/EA 7.9%; *P* = 0.914). There was one case of wound infection (2.6%) and one gastric retention (2.6%) in the LCC/IA group. While in the LAC/EA, we identified one wound infection(1.4%), one pulmonary infection(1.4%), one hematochezia (1.4%), three abdominal chyle leakage (4.1%), one bowel obstruction (1.4%) and one anastomotic leakage (1.4%). The patient experiencing postoperative adhesive ileus (CD Grade ≥ III) was reoperated with adhesiolysis, while the individual who developed mild anastomotic leakage (CD Grade ≥ III) was treated with anastomosis refashioning. Both recovered well after prompt surgical intervention. Other complications were improved with conservative management.

### Comparison of costs

In the LCC/IA group, surgical instruments cost approximately 2400 more RMB than the LAC/EA group both in the unmatched (25,945.8 ± 1,918.0 vs. 23,551.9 ± 2,665.5 RMB; *P* < 0.01) and matched team (25,945.8 ± 1943.8 vs. 23,560.3 ± 2758.7 RMB; *P* < 0.01). Expenses of anesthesia and intraoperative drugs were not significantly different between both groups. The total intraoperative costs were greater in the LCC/IA, with about 2,500 RMB additional charge per person, both before (30,843.8 ± 2400.9 vs. 28,395.8 ± 2724.7 RMB; *P* < 0.01) and after propensity score matching (30,843.8 ± 2433.1 vs. 28,375.8 ± 2820.9 RMB; *P* < 0.01).

Perioperative costs including diagnostic tools, ward and perioperative drugs did not reach statistical significance. However, in the LCC/IA group, ward charges were approximately 100 RMB lower and the numerical difference decreased by 70 RMB after PSM. The perioperative costs were on average 600 RMB less in the LCC/IA group, with the difference shrinking by 400 RMB in the matched data.

While overall hospital costs were not significantly different, patients underwent LCC/IA paid about 2000 RMB more compared to those who underwent LAC/EA. This may be attributed to the higher expenses of surgical instruments used for intracorporeal anastomosis than those used for extracorporeal anastomosis.

Further utilization and cost details are shown in Table 5.

**Table 5:** ^a^ In-hospital costs before and after propensity score matching

	Before propensity score matching			After propensity score matching		
	LCC/IA(*N* = 39)	LAC/EA(*N* = 74)	*P* value	LCC/IA(*N* = 38)	LAC/EA(*N* = 63)	*P* value
Intraoperative costs						
Surgical instruments	25,945.8 ± 1918.0	23,551.9 ± 2665.5	** < 0.01**	25,945.8 ± 1943.8	23,560.3 ± 2758.7	** < 0.0**1
Anesthesia	2,745.5 ± 334.7	2,673.3 ± 271.0	0.288	2,735.5 ± 339.2	2,660.6 ± 278.4	0.231
Intraoperative drugs	2,071.6 ± 538.0	2,195.2 ± 508.6	0.231	2,071.6 ± 545.3	2,184.9 ± 520.5	0.558
Total	30,843.8 ± 2400.9	28,395.8 ± 2,724.7	** < 0.01**	30,843.8 ± 2433.1	28,375.8 ± 2820.9	** < 0.01**
Perioperative costs						
Diagnostic tools	6,354.9 ± 2031.5	6,204.5 ± 2919.3	0.750	6,354.8 ± 2058.8	6,253.4 ± 2993.3	0.841
Ward	2,238.7 ± 733.9	2,353.6 ± 975.1	0.520	2,238.7 ± 743.8	2,306.8 ± 1014.9	0.720
Perioperative drugs	3,782.6 ± 2688.0	4,315.2 ± 3536.3	0.412	3,782.6 ± 2724.1	4,170.9 ± 3474.6	0.558
Total	12,376.2 ± 4038.9	12,873.3 ± 6112.5	0.648	12,376.1 ± 4093.1	12,731.1 ± 6176.5	0.754
Overall costs	43,220.0 ± 4954.0	41,269.2 ± 6685.9	0.112	43,219.9 ± 5020.6	41,106.8 ± 6867.7	0.102

Bold values indicate the significant differences (p < 0.05) between groups

^a^Costs regarding readmission/reoperation were not included

## Discussion

LCC/IA has been previously reported a year after laparoscopic colectomy was first employed [16]. The optimal way for fashioning anastomosis, particularly in the right colon, is still controversial [10, 17] [18]. Apart from technical challenges, concerns such as the risk of intraoperative contamination and tumor exposure have limited the clinical utilization of LCC/IA to a certain extent. What’s more, there have been few reports associated with the economic benefits of LCC/IA vs. LAC/EA. Thus, it is not clear which anastomosis technique would be more cost-effective.

The aim of our study is to compare the clinical outcomes in terms of operative and postoperative parameters, and the economic effectiveness between the two anastomosis techniques (LCC/IA vs. LAC/EA) in laparoscopic surgery for non-metastatic resectable colon cancer. Propensity score matching analyses were introduced to overcome the potential selection bias and data heterogeneity.

Advantages of LCC/IA regarding postoperative functional recovery include reduced mesenteric lacerations and bowel torsion during bowel exteriorization through a small laparotomy. Additionally, LCC/IA requires less mobilization within the abdominal cavity compared to LAC/EA. Therefore, the reduction of direct manipulation, less traction, and twisting with intestines could potentially minimize intraoperative bleeding and enhance bowel recovery. In accordance with previous studies and meta-analysis [18, 25], our finding indicates that LCC/IA is linked to earlier flatus passage and soft diet intake. Patients who underwent LCC/IA suffered less pain at POD3, though no significant difference was observed. This was slightly different from certain prior studies reporting a significantly lower VAS score associated with the LCC/IA technique [26, 27]. The reason for this difference may stem from variations in the location of laparotomy for bowel or specimen extraction across these articles; however, all patients in our LCC/IA cohort were chosen for supraumbilical incision for tumor extraction. Besides, mean postoperative LOS was nearly 1 day shorter in the LCC/IA group, averaging 5.6 days per person. While recent RCTs [22, 25] did not show the statistical difference, some observational reports and meta-analysis [18–20] have indicated such a distinction. It is worth noting that the retrospective nature of the design may introduce bias in drawing conclusions. The absence of enhanced recovery after surgery (ERAS) protocol in our perioperative management might have resulted in a longer duration of hospitalization.

Occurrence of 30-day mortality and 30-day postoperative complications were comparable between the two groups, in line with previous RCTs and meta-analysis [19, 20, 23, 24, 26, 28, 29]. In the LCC/IA group, one patient developed a superficial surgical wound infection (Grade I) that healed successfully with drainage and antibiotic therapy. Another patient, who did not start early ambulation, encountered functional gastric retention (Grade II) but was treated with drugs and gastrointestinal decompression with good recovery. Graded ≥ III complications such as bowel obstruction and anastomotic leakage were more frequent in the LAC/EA group in our study. Biondi et al. [30] speculated that LCC/IA could minimize bowel adhesion formation in the abdominal cavity, though the small bowel obstruction was not significantly different between the two surgical methods. So we considered that both anastomosis techniques were safe.

In terms of pathological outcomes, LCC/IA was associated with significantly more dissected lymph nodes; however, the statistical difference disappeared after PSM. The result aligned with previous studies demonstrating higher amount of harvested lymph nodes with the IA method [31, 32]. This finding reflected the fact that LCC/IA could offer better visualization of the bowel to perform complete exposure of the mesenteric base and vascular ligation under a fully laparoscopic view.

Longer operative time was found in the LCC/IA group, likely due to the technological challenge and longer learning curve. Although several studies have noted additional time required for creating IA [8, 23, 26, 32, 33], we assume that this duration will gradually decrease as surgical expertise grows and familiarity with the technique increases.

As a potential advantage, LCC/IA enables the selection of specimen extraction sites easier [22]. Studies have shown a lower risk of incisional hernia with off-midline (Pfannenstiel or transverse) compared to midline incision [34, 36]. As a result, more surgeons prefer off-midline incision to deliver specimens [37]. On the other hand, the extraction incision of LAC/EA is often confined by the planned anastomosis location and mesocolon anatomy. Therefore, midline incisions were more frequently used with relatively longer lengths in comparison to LCC/IA [21]. A few articles have reported that using a Pfannenstiel incision to extract specimens in LCC/IA led to a much shorter incision length than in LAC/EA [27, 38, 39]. In our practice, we opted for a supraumbilical incision as the specimen extraction site in both groups; however, the incision length was still significantly shorter in the LCC/IA cohort. This may be attributed to a relatively larger incision to exteriorize the bowel when fashioning extracorporeal anastomosis. Smaller incision provided a better cosmetic outcome and lower level of postoperative pain, compatible with previous conclusions [22, 40]. Although no incisional hernia was observed in our study, the relationship between incisional hernia occurrence and the location or length of the extraction site still lacks clarity. Further investigation with prolonged follow-up period and varied minilaparotomy for specimen extraction might provide additional insights.

A standardized IA process remains elusive. Mechanical anastomosis, including triangular, overlap and π anastomosis, are commonly used. Despite LCC/IA has been proven to be a priority regarding less intra- and postoperative complications, a recent large-scale retrospective analysis presented contradictory results [32]. Sun et al. noted an increased risk of abdominal infection and mild postoperative complications (Grade I-II) associated with IA. They pointed out that this might due to the fact that surgeons repeatedly used liner staplers for side-to-side configuration and lengthened the main working port for specimen extraction instead of selecting another site. To improve the safety and quality of IA and mitigate complications, we implemented the previously described UEEA approach. This approach could simplify the IA steps and make them modular and procedural, thus promoting homogeneity of the IA techniques, as well as reducing tissue trauma in the anastomic region and alleviating the financial burden on patients [11].

The economic benefits of LCC/IA versus LAC/EA after radical resection for colon cancer have been infrequently reported. RCT Torino trial [41] indicated a higher cost in the intracorporeal anastomosis (IIA) group compared to the extracorporeal anastomosis (EIA) group following laparoscopic right colectomy (LRC); though the difference did not achieve statistical significance. However, the IIA group incurred significant intraoperative additional costs (*P* < 0.001). The researchers suggested these results were possibly due to variations in stapler and cartridge types and a higher proportion of hand-sewn anastomosis in the IIA cohort. Similarly, a small-sample retrospective study comparing extracorporeal anastomosis (ECA) and intracorporeal anastomosis (ICA) after LRC [42] found comparable total costs. ICA required higher expenses for surgical tools but shorter hospital stays, while ECA had lower surgical supply charges but a longer recovery period. These analyses did not definitively favor one anastomotic technique over the other with regard to cost-effectiveness. In a population-based gastric cancer research, Shinohara et al. [43] found that the increased operation cost of totally laparoscopic distal gastrectomy (TLDG) was offset by the decreased costs associated with longer hospitalization periods.

Our study, consistent with previous cost analysis, revealed significantly higher surgical instrument and intraoperative costs in the LCC/IA group; however overall costs and other financial outcomes were comparable between the two cohorts. Indeed, a totally mechanical side-to-side anastomosis with an endoscopic linear stapler was utilized in the LCC/IA, while approximately 20% of patients who underwent LAC/EA received a hemi hand-sewn anastomosis. And the stapler used for IA was more costly than that for EA. Despite the increased surgical expenses, LCC/IA demonstrated superior postoperative recovery (faster bowel function restoration, pain alleviation, and shorter hospital duration). Thus, we speculated the additional surgical cost was offset by less medication use and shorter postoperative LOS. Consequently, LCC/IA may provide patients with potential advantages both in safety and hospital charges. We expect the potential benefits will become more dramatic as surgical technique is involved and surgical proficiency increases.

Several limitations should be pointed out in our study. Firstly, the follow-up period was insufficient for a comprehensive analysis of oncological outcomes between the two groups. Secondly, the retrospective and non-randomized design with a small sample size in a single institution may have resulted in underpowered and under-generalized findings. However, the strengths should not be neglected. One is performing PSM to establish two homogeneous groups treated with standardized surgical procedures and perioperative management. In addition, this was the first cost analysis regarding intraoperative and postoperative outcomes following both right and left hemicolectomy by LCC/IA or LAC/EA technique. Furthermore, our team innovatively employed the UEEA approach to fashion intracorporeal anastomosis.

A prospective multi-center study with a larger population is recommended for further analysis. Future researches are anticipated to help determine the most economically efficient anastomosis technique and assist surgeons in making appropriate surgical decisions, while also providing patients with comprehensive survival information.

## Conclusion

In comparison to LAC/EA, LCC/IA was associated with higher surgical costs but equivalent overall charges. It offers benefits such as reduced intraoperative blood loss, improved cosmetic outcome, and quicker postoperative functional recovery. These advantages are anticipated to have a positive impact both on safety and hospital charges. As a consequence, we suggest LCC/IA as a feasible, safe, and cost-effective surgical option for patients with non-metastatic resectable colon cancer.

## Data Availability

The data are not publicly available due to ethical restrictions.
